# Supplementary material for: Chromothripsis during telomere crisis is independent of NHEJ, and consistent with a replicative origin
Source: Genome Res. 2019 May;29(5):737–49. doi: 10.1101/gr.240705.118 (PMC6499312; doi:10.1101/gr.240705.118)
Supplement: Supplemental Material [file supp_gr.240705.118_Supplemental_file_1.zip › contigs/annotated_contigs/DB99/contig.2.DB99_length_685_mean_cov_7.02189781022.docx]

**DB99_length_685_mean_cov_7.02189781022**

```
AAAAAGACTTAGACTCCCACACAATAATTGTAGGAGACTTTAACACCCCACTGTAGGTATTAGACCTATCAATGAGACAGAAAATTAAC
 >chrX:121135465-121135842 - E=4e-212
AAGGATATGCAGGACTTGAACTCAGCACTGGATCAAGTGGACCTGGTAGACATATACAGAACTCTCTACCCCAAATCAACAGAATATAC

ATTCTTCTCAGTGCCACACGACGCTTATTCTAAAATCAACAACATAAATGGAAGTAAAACACTCCTCAGCAAATGCAAAAGAACTGAAA

TCATAACAGTCTCTCAGACACAATGAAATCAAATCAGAACTCAGGATTAAGAAACTCACTCAAAACCACACAACTACATGGAAATTGAA

CAACCTGCCCCTGAATGATGT|TA|GATTGCCATTCTAACTGGTGTGAGATGGTATCTCATTGTGGTTTTGATTTGCATTTCTCTGATG
                        >chrX:121631979-121632283 - E=4e-165
GCCAGTGATGATGAGCATTTTTTCATGTGTCTTTTGGCTGCATAAATGTCTTCTTTTGAGAAGTGTCTGTTCATATCCTTTGCCCACTT

TTTGATGGGGTTGTTTTTTTCTTTTAAATTTGTTTGAGTTCATTGTAGATTCTGGGTATTAGCCCTTTGTCAGATGAGTAGGTTGTGAA

AATTTTCTCCCATGTTGTAGGTTGCCTGTTCACTCTGATGGTAGTTTCTTTTGCTGGGCAGA|GG
```
